# 3D Printing of Cytocompatible Water-Based Light-Cured Polyurethane with Hyaluronic Acid for Cartilage Tissue Engineering Applications

**DOI:** 10.3390/ma10020136

**Published:** 2017-02-08

**Authors:** Ming-You Shie, Wen-Ching Chang, Li-Ju Wei, Yu-Hsin Huang, Chien-Han Chen, Cheng-Ting Shih, Yi-Wen Chen, Yu-Fang Shen

**Affiliations:** 1 3D Printing Medical Research Center, China Medical University Hospital, China Medical University, Taichung 40447, Taiwan; eviltacasi@gmail.com (M.-Y.S.); Jayla11218@gmail.com (W.-C.C.); lijuwei928@gmail.com (L.-J.W.); yhhuang@dragon.nchu.edu.tw (Y.-H.H.); songofdeath2121@gmail.com (C.-T.S.); 2 School of Dentistry, China Medical University, Taichung 40447, Taiwan; 3 School of Medicine, College of Medicine, China Medical University, Taichung 40447, Taiwan; u102001701@cmu.edu.tw; 4 Graduate Institute of Biomedical Sciences, China Medical University, Taichung 40447, Taiwan; 5 Department of Bioinformatics and Medical Engineering, Asia University, Taichung 40447, Taiwan

**Keywords:** water-based polyurethane, hyaluronic acid, cartilage tissue engineering, scaffold

## Abstract

Diseases in articular cartilages have affected millions of people globally. Although the biochemical and cellular composition of articular cartilages is relatively simple, there is a limitation in the self-repair ability of the cartilage. Therefore, developing strategies for cartilage repair is very important. Here, we report on a new liquid resin preparation process of water-based polyurethane based photosensitive materials with hyaluronic acid with application of the materials for 3D printed customized cartilage scaffolds. The scaffold has high cytocompatibility and is one that closely mimics the mechanical properties of articular cartilages. It is suitable for culturing human Wharton’s jelly mesenchymal stem cells (hWJMSCs) and the cells in this case showed an excellent chondrogenic differentiation capacity. We consider that the 3D printing hybrid scaffolds may have potential in customized tissue engineering and also facilitate the development of cartilage tissue engineering.

## 1. Introduction

Cartilage reconstruction is an important topic in regenerative medicine [1,2,3,4,5,6,7]. Because of the avascularity of articular cartilage, the limited proliferation of mature chondrocytes and less migration of chondrocytes surrounded by the extracellular matrix, the regeneration of articular cartilage is considered difficult in comparison to other tissue [2]. Many approaches such as microfracture, abrasion arthroplasty, osteochondral autologous transfer, and autologous chondrocyte implantation have been applied for cartilage reconstruction. Microfracture is suitable for small cartilage injuries. Brasion arthroplasty may cause thermal necrosis. The recipients of allogenic cartilages may suffer body reactions and infections. Besides, autografts have problems related to limited availability. Therefore, there is still no ideal and perfect approach for the reconstruction of critical articular cartilage defects and there are many problems that need to be resolved [8].

Currently, three-dimensional (3D) printing is considered an effective and high potential technology to revolutionize the field of regenerative medicine and tissue engineering. Previous studies also reported 3D printing as being a useful method to fabricate scaffolds for cartilage tissue engineering [9,10]. In 2012, Xu et al. [9] used a novel multi-head deposition system to fabricate hybrid scaffolds for cartilage tissue engineering applications. In 2014, Hung et al. [10] established a water-based platform technology for 3D-printed cartilage scaffold fabrication with liquid-frozen deposition manufacturing (LFDM). In 2015, Markstedt’s group [11] constructed 3D cartilage scaffolds with a bio-ink composed of nanofibrillated cellulose and alginate by electromagnetic jet technology. Although their studies demonstrated 3D printing can provide a solution for cartilage tissue engineering, the mechanical properties of the printed scaffolds were weaker than natural articular cartilage and the printing resolution needed to be improved. 

3D printing supplies a lot of advantages for medical applications, containing high precision, fast fabrication and customized production. Many 3D printing methods including fused deposition manufacturing (FDM) [12], LFDM [13], selective laser sintering (SLS) [14,15], power bed and inkjet head 3D printing (PIP) [16], stereolithography (SLA) [17], and digital light processing (DLP) [18] have been applied for tissue engineering scaffold fabrication. Although FDM and LFDM technologies can provide a low manufacturing cost and a simple manufacturing process, the resolution is limited on a *Z*-axis. The problems of SLS and PIP technologies are the large amounts of waste production. SLA and DLP have higher vertical resolution [19] than other 3D printing technologies, but most light-curable polymers are dissolved in organic solvents which have low biocompatibility [20]. Therefore, researchers have focused on the development of light-curable and highly biocompatible materials for SLA and DLP in recent years [21,22,23], such as poly(ε-caprolactone)-based materials, poly(d,l-lactide) resins, and poly(ethylene glycol)/poly(d,l-lactide)-based resins.

An ideal scaffold for tissue engineering is biocompatible and biodegradable and owns a desired tissue shape and porous structure for providing a nutrient and metabolic transporting pathway. DLP technology can satisfy the requirements of printing 3D scaffolds which have specific shape and porous structure, however the materials for DLP technology are the key to decide if the printed scaffolds are biocompatible and biodegradable. Biodegradable materials can be gradually degraded by biologic fluid in vivo or by microorganisms in the environment [24]. Previous studies reported tissue engineering scaffolds can be fabricated by biodegradable materials including poly(ε-caprolactone) (PCL), polylactic acid (PLA), polyglycolic acid (PGA), and polylactic-co-glycolic acid (PLGA) [25]. However, the compressive strength and Young’s modulus of these materials are not similar to living tissues and some of these materials often need to be dissolved in highly toxic solvents [26,27]. Biostable polyurethanes are considered to have good biocompatibility and mechanical properties for long term medical implants including vascular grafts and cardiac pacemakers. Polyurethanes are generally synthesized through polycondensation reaction of di-isocyanates with amines and/alcohols [28]. In this study, polyurethanes containing aliphatic polyesters which allow for biodegradation were applied. Besides, the polyurethanes are water-based light-cured polyurethanes which could be applied in DLP technology and have good biocompatibility. 

Water-based 3D printing of photosensitive materials for customized cartilage tissue engineering with DLP technology was developed in this study. Water-based light-cured polyurethane is a non-toxic and environmentally friendly material, but it has not yet been applied in DLP technology for 3D printing. Here, we report a new liquid resin preparation process for water-based polyurethane based photosensitive materials with hyaluronic acid (HA) which has been reported to promote cartilage repair, with application of the materials for 3D printed customized cartilage scaffolds. The scaffold has high biocompatibility and is one that closely mimics the mechanical properties of articular cartilages. Furthermore, it can be designed by three-dimensional reconstruction and is similar to the shape of the cartilage defect of the recipient site in order to provide the most effective way for cartilage tissue reconstruction, as well as also facilitating longer term the development of cartilage tissue engineering.

## 2. Results and Discussion

### 2.1. Fabrication of Customized Scaffolds

Organic solvents such as DMF (dimethylformamide) are involved in traditional polyurethane (PU) polymerization to regulate PU viscosity to make PU coating easier. In order to substitute traditional solvent-based PU which uses volatile organic compounds (VOC), water-based PU have been developed in recent years [29]. Although water-based light-cured PU is a non-toxic and environmentally friendly material, it has been used for coating previously but was not applied in DLP and SLA technologies for 3D printing directly. During the curing process, the water in water-based light-cured PU resins must be removed by heat or short wave infrared. Besides, the water-based light-cured PU resins often present a solidified or over-sticky state after water removal. 

**Figure 1:**
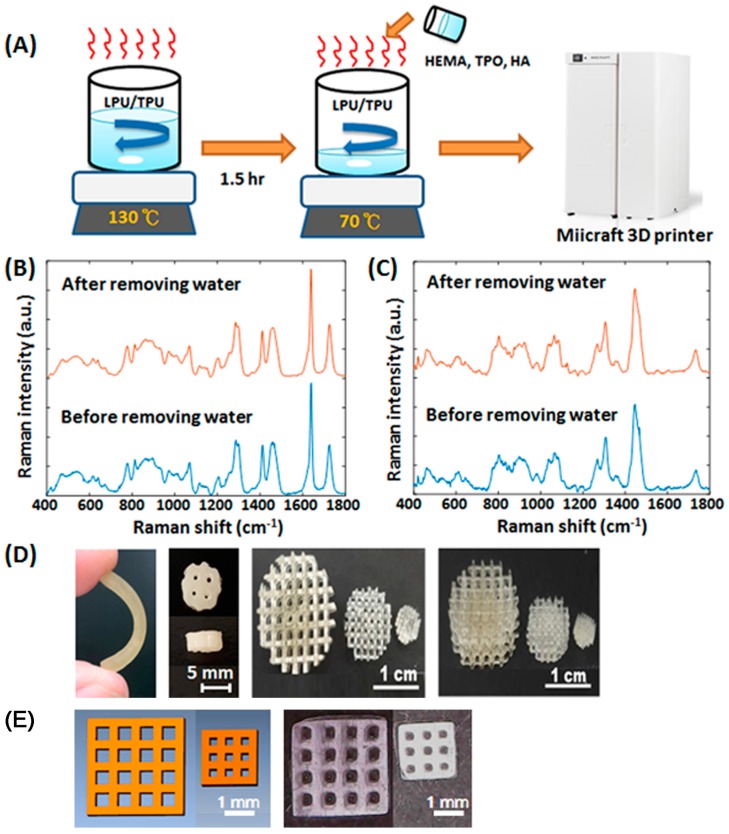
(**A**) The schematics of the manufacturing process of the water-based polyurethane based photosensitive materials; The Raman spectra of the (**B**) water-based light-cured polyurethanes and (**C**) water-based thermoplastic polyurethanes with or without water removal processes; (**D**) The images of the printed scaffolds; (**E**) The images of the designed (left) and printed (right) porous lattice structures.

The viscosity of the materials for DLP based 3D printers is a key factor which can affect the printing resolution and printing results [30]. Here, we report a new liquid resin preparation process for water-based polyurethane based photosensitive materials (Figure 1A). The water-based polyurethanes were heated and stirred at high speed to remove the water, and then hydroxyethylmethacrylate (HEMA) to adjust the viscosity and photoinitiators for light curing were added. There is no significant difference in the Raman spectra of the water-based polyurethanes between the with or without water removing processes (Figure 1B,C). Besides, scaffolds with various shapes were fabricated by DLP technology to confirm the printing resolution and customized potential of the materials (Figure 1D,E). The designed lattice widths in the 4 × 4 and 3 × 3 porous lattice structures were 0.5 and 0.3 mm (Figure 1E). The average lattice widths in the printed porous lattice structures were 0.517 and 0.306 mm, respectively. The percent errors of the printed lattice width were lower than 4% compared to the original design. These results indicated our materials can be printed out in the shape of the design and have customized potential.

### 2.2. Characterization of Water-Based Light-Cured PU/TPU Specimens

**Figure 2:**
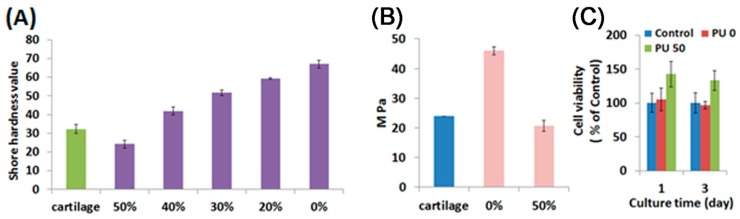
The physical properties and cell viability of water-based polyurethane (PU) based photosensitive materials with different proportions of the water-based thermoplastic polyurethane. (**A**) Shore hardness values and (**B**) Young’s modulus of the water-based polyurethane based composites compared with articular cartilage; (**C**) Cell viability of hWJMSCs cultured on the water-based polyurethane based composites with 0% or 50% of the water-based thermoplastic polyurethane for 1 and 3 days. Values in (**A**–**C**) represent mean and SD (*n* = 3).

Water-based polyurethanes are of different types, such as water-based light-cured polyurethanes, water-based thermoplastic polyurethanes, and water-based thermosetting polyurethanes. In this study, the water-based light-cured polyurethane and the water-based thermoplastic polyurethane were mixed and heated together to remove water and to form hybrid materials. By using different proportions of the water-based light-cured PU and the water-based thermoplastic polyurethane, the hybrid specimens showed different hardness and different Young’s modulus values (Figure 2A,B). When increasing the concentration of the water-based TPU, the hardness and Young’s modulus decreased significantly. The Young’s modulus of the articular cartilage according to previous studies is about 24 MPa [31]. When adding specific proportions of the water-based TPU, the physical properties of the materials were similar to articular cartilage. Although previous reports [10,32] have also developed 3D printing materials to fabricate the tissue-engineered cartilage constructs, their compressive strength and Young’s modulus were much lower than natural articular cartilage. In addition, the cell viability of hWJMSCs cultured on the water-based polyurethane based composites with 0% or 50% of the water-based TPU for one and three days was evaluated (Figure 2C). The hWJMSCs cultured on 0% or 50% water-based thermoplastic polyurethane composites had similar or higher cell viability compared with the normal tissue culture plates (Control). In 2012, Chu et al. [33] also reported that the attachment and migration of WJMSCs can be promoted by PU. These results demonstrated the polyurethane based composites have high biocompatibility.

### 2.3. Characterization of Water-Based Light-Cured PU/HA Scaffolds

HA is an important component of articular cartilage. It can link aggrecan molecules to large proteoglycans and be a lubricant in joints [34]. Previous studies reported that HA can facilitate cell migration and viability [35] and may promote the chondrogenic differentiation of mesenchymal stem cells (MSCs) [36,37]. In 2016, Gobbi’s group showed that HA based scaffolds with activated bone marrow-derived MSCs can provide better effects of cartilage reconstruction than microfracture and lead to successful medium-term outcomes [38]. Therefore, the HA based scaffolds with stem cells may provide great potential for the development of cartilage repair. 

**Figure 3:**
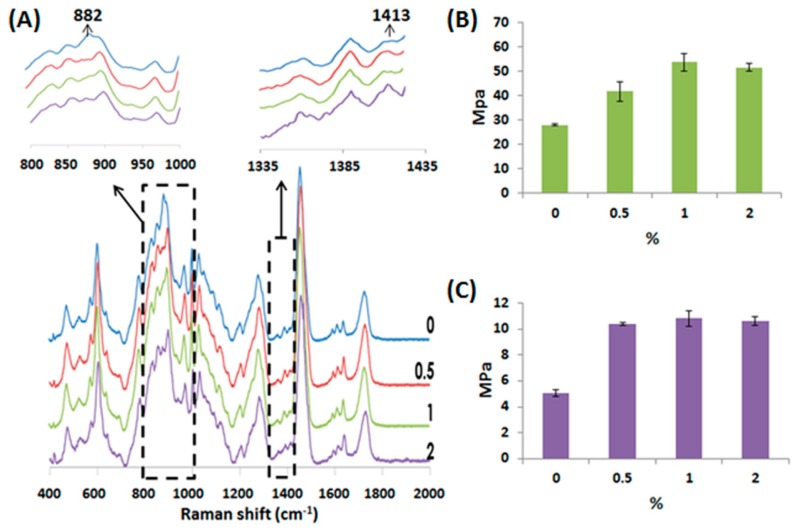
(**A**) Raman spectra of the PU/HA hybrid scaffolds with different hyaluronic acid (HA) concentration; The (**B**) Young’s modulus and (**C**) diametral tensile strength values of the PU/HA hybrid scaffolds.

In this study, HA was added in the water based polyurethane composites to fabricate water-based light-cured PU/HA 3D hybrid scaffolds by DLP technology. Figure 3A shows the Raman spectra of the PU/HA hybrid scaffolds with different HA concentrations. The Raman spectra of the PU/HA hybrid scaffolds with different HA concentrations were very similar. When increasing the HA concentration, the height of the 1413 cm^−1^ band increased slightly. The width of the 1413 cm^−1^ band, related to the symmetrical vibration of the COO^−^ group of the glucuronate residue, was used to identify HA [39].

The Young’s modulus and diametral tensile strength (DTS) values of the PU/HA hybrid scaffolds are shown in Figure 3B,C. The Young’s modulus and DTS values had significant increases in the strength in the scaffolds with HA. We suggest that the added HA could react with PU to form a tighter chemical structure. In addition, the Raman spectra of the PU/HA hybrid scaffolds showed that the height of the 882 cm^−1^ band of the scaffolds with HA decreased slightly compared to the scaffolds without HA (Figure 3A) and the peak at 882 cm^−1^ was attributed to C–C–O vibrations of PU. These results indicated that HA may interact with PU to cause the height of the 882 cm^−1^ band to decrease and to lead the scaffold strength to increase.

**Figure 4:**
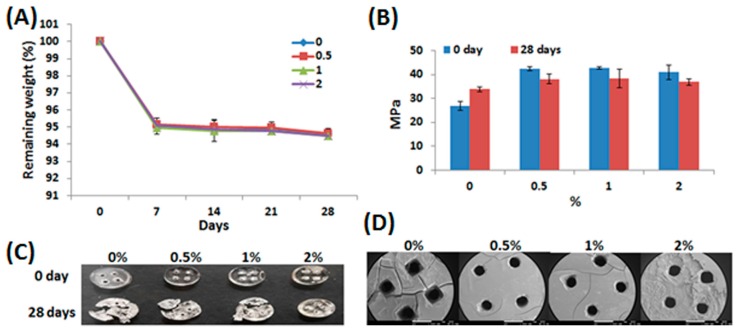
The mechanical properties and degradation rate of the PU/HA hybrid scaffolds. (**A**) The degradation profile of the PU/HA hybrid scaffolds with different HA concentration, expressed as percent remaining weight; The (**B**) Young’s modulus and (**C**) images of the PU/HA hybrid scaffolds after compressing tests for 0- and 28-day degradation tests; (**D**) The SEM images of the PU/HA hybrid scaffolds after 28-day degradation tests. The scale bar is 1 mm.

Figure 4A shows the degradation results of the PU/HA hybrid scaffolds for 7, 14, 21, and 28 days in phosphate buffered saline (PBS) at 37 °C. The degradation results of all the scaffolds were almost the same. All PU/HA hybrid scaffolds displayed a rapid initial weight loss in 7 days. After 28 days, the weight loss measured for all PU/HA hybrid scaffolds was about 94.5%. In 2011, Tan’s group reported HA-HA hydrogels showed a fast weight loss and fully degraded in 10 days [40]. However, the degradation results of the scaffolds with or without HA were almost the same in our study. We suggest that the amount of HA added was too low to allow the degradation rate to be affected. Although polyurethanes containing aliphatic polyesters are biodegradable materials, the PU/HA hybrid scaffolds exhibited slow degradation rates before 28 days. A previous report [41] pointed out that the scaffold degradation was correlated to the chemical design of the original polymer and slow degradation rates of aliphatic polyesters before 30 days were also observed. The Young’s modulus values of the PU/HA hybrid scaffolds were also evaluated after 28 days (Figure 4B). The Young’s modulus of the scaffolds with HA decreased slightly after 28 days, but the Young’s modulus of the scaffolds without HA increased slightly. Figure 4C shows the images of the PU/HA hybrid scaffolds after compressing tests. All the PU/HA hybrid scaffolds without degradation tests only presented deformation after compressing, but the phenomenon of fragmentation was caused in the scaffolds containing 0%–1% HA with 28-day degradation tests. It is noteworthy that the PU/HA hybrid scaffolds with 2% HA only presented the phenomenon of deformation after compressing. The SEM images of 28-day degradation tests showed that the phenomenon of crack formation reduced gradually with increasing HA concentration (Figure 4D). These results indicate that the addition of HA can prevent crack formation of scaffolds during the degradation process and may facilitate stable degradation of the scaffolds.

### 2.4. Adhesion, Proliferation and Chondrogenic Differentiation of Cells Cultured on Water-Based Light-Cured PU/HA Scaffolds

**Figure 5:**
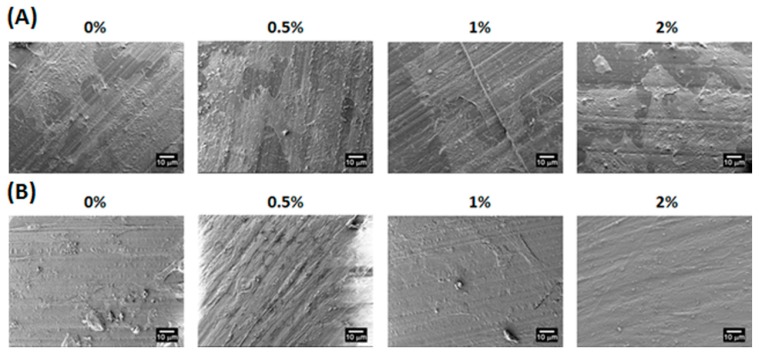
The scanning electron microscopy (SEM) micrographs of hWJMSCs cultured on the PU/HA hybrid scaffolds with different HA concentration for (**A**) 4 h and (**B**) 3 days.

**Figure 6:**
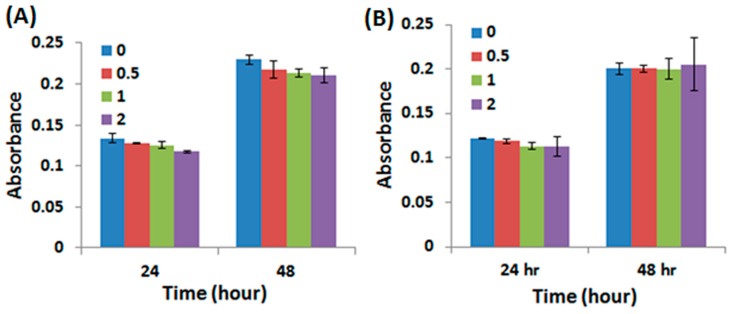
The cell proliferation of (**A**) WJMSCs and (**B**) chondrocytes cultured on the PU/HA hybrid scaffolds for 24 and 48 h.

**Figure 7:**
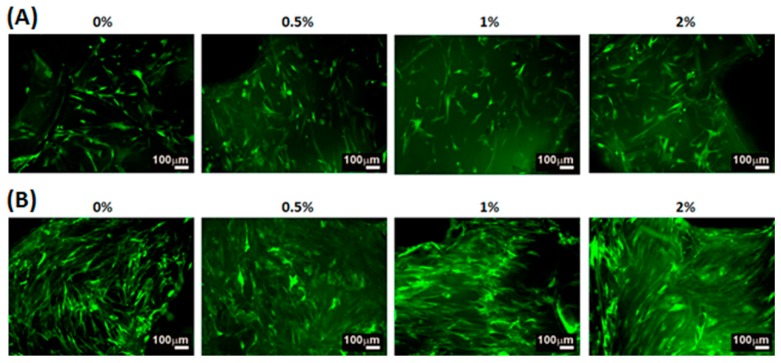
The fluorescent images of hWJMSCs cultured on the PU/HA hybrid scaffolds for (**A**) 1 and (**B**) 5 days.

The cell morphology of WJMSCs cultured on the PU/HA hybrid scaffolds for 4 h and 3 days were examined by the SEM images (Figure 5). The cells on the all PU/HA hybrid scaffolds for 4 h displayed flat and presented intact, well-defined morphology. This result demonstrated that the cells can adhere on the scaffolds very well and that the scaffolds might provide a good adhesion environment for the cells. Besides, the SEM images of the cells on all the PU/HA hybrid scaffolds for 3 days showed that the cells still presented good cell morphology and the number of cells increased. The cell proliferation of WJMSCs and chondrocytes cultured on the PU/HA hybrid scaffolds was evaluated by the PrestoBlue^®^ assay (Figure 6). In addition, the fluorescent images showed the WJMSCs covered all the PU/HA hybrid scaffolds after 5 days incubation (Figure 7). These results indicated that cells incubated on PU/HA hybrid scaffolds can have good proliferation ability and the scaffolds have good biocompatibility.

**Figure 8:**
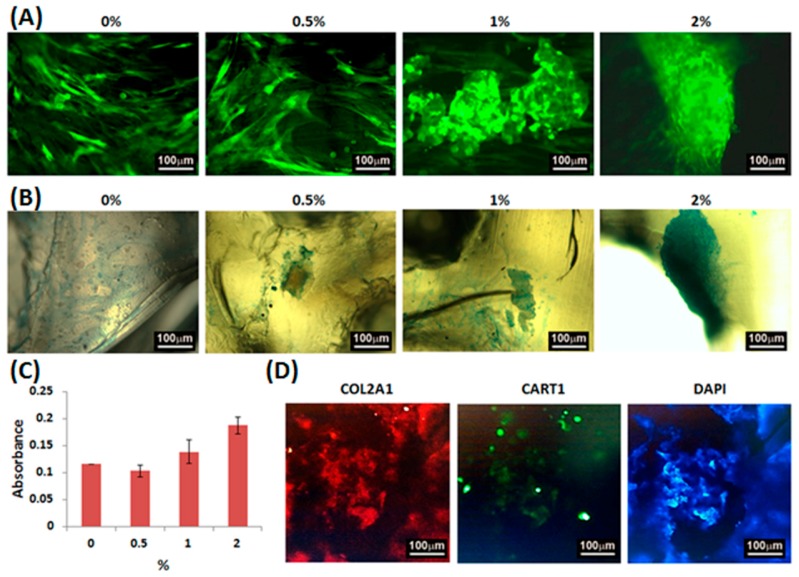
The (**A**) fluorescent and (**B**) Alcian blue staining images and the (**C**) GAG contents of the micromass cultures of WJMSCs cultured on PU/HA hybrid scaffolds for 1 day; (**D**) The immunofluorescence staining images of nuclei (blue), COL2A1 (red) and CART1 (green) for the micromass cultures of WJMSCs cultured on PU/HA hybrid scaffolds with 2% HA for 1 day.

In order to investigate the chondrogenic differentiation effect of the scaffolds, the fluorescent and Alcian blue staining images and the glycosaminoglycan (GAG) contents of the micromass cultures of WJMSCs cultured on the PU/HA hybrid scaffolds for 1 day were evaluated (Figure 8A–C). The fluorescent images showed that the phenomenon of cell aggregation increased as the HA concentration increased. Besides, the color of the Alcian blue staining ranged from light to deep blue and the GAG contents increased as the HA concentration increased. Furthermore, the immunofluorescence staining images (Figure 8D) showed that the WJMSCs cultured on the PU/HA hybrid scaffolds with 2% HA and without adding chondrogenic differentiation medium can express collagen type II and cartilage homeoprotein 1 (CART1) which are the markers of chondrogenic differentiation. These results indicated that the PU/HA hybrid scaffolds containing higher HA concentration might stimulate chondrogenic differentiation. In 2015, Nalluri’s group [42] reported that hydrophilic polyurethanes can show a biphasic structure and present a gel-like architecture to provide a compatible synthetic matrix for chondrogenic differentiation of MSCs. In 2016, Huang’s group showed that the GAG secretion by chondrocytes in water-based polyurethane 3D printed scaffolds was greater than that in PLGA scaffolds [10]. Although they also used water-based polyurethane to manufacture 3D printed scaffolds, their scaffolds were fabricated by LFDM technology which has lower printing resolution. Besides, Yoo et al. [43] and Fan et al. [44] reported the PLGA scaffolds containing hyaluronic acid presented greater chondrogenic differentiation ability. These previous results indicated hydrophilic polyurethanes have the potential to promote chondrogenic differentiation and the chondrogenic differentiation ability can be improved by adding hyaluronic acid; similar to our results. In this study, we provided a new liquid resin preparation process of water-based polyurethane based photosensitive materials with HA for DLP technology, so that the printing resolution was higher than the previous study [10] and the printed 3D scaffolds also have good biocompatibility and promote chondrogenic differentiation. Furthermore, several reports [45,46,47] show shape memory polymers fabricated with 3D printing technology bring about the possibility to realize 4D printing. The polyurethanes are also shape memory polymers and have the potential for 4D printing. Therefore, we also expect to develop 4D printing in the future on the basis of our current research for cartilage tissue engineering.

## 3. Materials and Methods

### 3.1. The Preparation of Water-Based Polyurethane Based Composites

Water-based light-cured polyurethanes (LUX 260) and water-based thermoplastic polyurethanes (U 2101) were purchased from Alberdingk Boley, Krefeld, Germany. Different proportions of the water-based thermoplastic polyurethanes (Liquid, Solid Content 50%) were added to the water-based light-cured polyurethanes (Liquid, Solid Content 40%). The hybrid materials containing 0%, 10%, 20%, 30%, 40% or 50% water-based thermoplastic polyurethanes were heated at 130 °C and stirred at high speed for 1.5 h to remove the water. Amounts of 1.5% 2,4,6-trimethylbenzoyl-diphenyl-phosphineoxide (TPO) photoininitiators (Ciba, Switzerland) and 0%, 0.5%, 1% or 2% 1900 kD HA (Suvenyl, Chugai Pharmaceutical, Tokyo, Japan) were dissolved in 2-Hydroxylethyl methacrylate (HEMA) (Sigma-Aldrich, St. Louis, MO, USA), and then were added to the light curing waterbone polyurethane composites to mix at 70 °C for 3D printing.

### 3.2. Graft Fabrication

All test objects and scaffolds were designed through SolidWorks (Dassault Systemes SolidWorks Corp., Waltham, MA, USA) and fabricated by a MiiCraft high resolution home DLP 3D printer (Young Optics Inc., Hsinchu, Taiwan). The mode of fabrication was blue light digital stereolithography to cure individual 100 μm layers of at 20 s exposure. For mechanical properties of the water-based light-cured PU/TPU specimens, the samples were printed with thickness of 3 mm and a diameter of 6 mm. In addition, the water-based light-cured PU/HA scaffolds were printed with thickness of 3 mm, a diameter of 6 mm and four rectangular holes (1 mm × 1 mm × 3 mm). The uncured materials were washed off and the scaffolds were post-cured under UV light, yielding the fully cured scaffolds. The cured scaffolds were washed again for 3D cell culture.

### 3.3. Printing Accuracy Analysis

Photographs of the structures were taken using a digital microscope with a pixel size of 0.265 × 0.265 mm^2^. Horizontal and vertical profiles were then acquired from the photographs to identify the edges of the holes. The width of each hole in the structures was calculated as the pixel distance between the edges of the hole times the pixel size. The designed lattice widths in the 4 × 4 and 3 × 3 porous lattice structures were 0.5 and 0.3 mm. The mean lattice width was calculated by averaging the width of all holes and was compared with the original design. 

### 3.4. Mechanical Properties 

The hardness of the water-based polyurethane based composites was determined by the shore hardness tester (Teclock Corp., Nagano, Japan). The mechanical properties were examined using an EZ-Test machine (Shimadzu, Kyoto, Japan) with a 500 N load cell at a loading rate of 1 mm/min. Young’s modulus was calculated from the linear region in stress-strain curve using a theoretical model.

### 3.5. Raman Spectroscopy

Raman spectra were collected by a portable i-Raman system (B&W Tek, Newark, DE, USA) with an accessional software BwRam1.1 (B&W Tek, Newark, DE, USA). The same spectral region chosen for the standard normal variant (SNV) transformation is needed [48] with differences of focus depth or sample volume.

### 3.6. Degradation In Vitro 

The degradation of the PU/HA hybrid scaffolds was examined in phosphate buffered saline (PBS) at 37 °C for 7, 14, 21, and 28 days. The remaining weight of the scaffolds was calculated by the following equation: remaining weight (%) = *W*_f_/*W*_i_ × 100% [49]. *W*_i_ was the initial weight of the scaffolds. *W*_f_ was the weight of the scaffolds which were rinsed with distilled deionized water and dried after the 7-, 14-, 21-, or 28-day degradation test.

### 3.7. Cell Viability

Approximately 10 thousand WJMSCs and chondrocytes were directly seeded over each scaffold for 1 and 3 days. Cell cultures were maintained at 37 °C in a 5% CO_2_ atmosphere. The cell viability was determined by the PrestoBlue^®^ (Invitrogen, Grand Island, NY, USA) assay. The values of the absorbance were examined in a multi-well spectrophotometer (Hitachi, Tokyo, Japan) at 570 nm with a reference wavelength of 600 nm.

### 3.8. Cell Morphology

After 4 h and 3 days of cell culture, the scaffolds with WJMSCs were washed with cold PBS and fixed by 1.5% glutaraldehyde (Sigma-Aldrich, St. Louis, MO, USA). After 2 h, the scaffolds were dehydrated through a graded ethanol series for 20 min at each concentration and dried with liquid CO_2_ by a critical point dryer device (LADD 28000, LADD, Williston, VT, USA). The dried scaffolds were mounted on stubs, coated with gold particles, and examined by scanning electron microscopy (JEOL JSM-7401F, Tokyo, Japan).

### 3.9. Fluorescent Images

The stable enhanced green fluorescent protein (EGFP) WJMSCs which were established by the Retro-XTM Universal Packaging System (Clontech Laboratories Inc., Mountain View, CA, USA) were cultured on the PU/HA hybrid scaffolds. After 1 and 5 days, the scaffolds with the stable cells were washed with PBS and the fluorescent images were investigated by a Zeiss Axioskop2 fluorescent microscope (Carl Zeiss, Thornwood, NY, USA).

### 3.10. Micromass Culture

The micromass culture technique was modified from Ahrens’s group [50]. The cell solution of 1.6 × 10^7^ viable cells/mL was prepared. Droplets of 5-µL of cell solution were seeded to generate micromass cultures on the scaffolds for fluorescent images, Alcian blue staining and analysis, and immunofluorescence staining.

### 3.11. Alcian Blue Staining and Analysis

The hWJMSCs were grown on the PU/HA hybrid scaffolds and without adding chondrogenic differentiation medium. After 24 h, the scaffolds with hWJMSCs were fixed with 4% formaldehyde, sulfuric acid for 30 min, and then stained with 10 mg/mL Alcian blue solution for 3 h. The Alcian blue staining images were obtained by a Zeiss Axioskop2 microscope (Carl Zeiss, Thornwood, NY, USA). Besides, the GAG-Alcian blue complexes can be dissociated and dissolved in a 4 M guanidine-HCl/propanol mixture after staining. The values of the absorbance were examined in a multi-well spectrophotometer (Hitachi, Tokyo, Japan) at 600 nm.

### 3.12. Immunofluorescence Staining

The hWJMSCs were grown on the PU/HA hybrid scaffolds with 2% HA and without adding chondrogenic differentiation medium. After 24 h, the scaffolds with hWJMSCs were fixed, and immunostained with anti-COL2A1 (rabbit), anti-CART1 (mouse), and then with anti-rabbit conjugated tetramethylrhodamine (TRITC), anti-mouse conjugated fluorescein isothiocyanate (FITC) and with 4′,6-diamidino-2-phenylindole (DAPI). Immunofluorescence images were examined by using a white light laser confocal microscope Leica TCS SP8 X (Leica Microsystems, Heidelberg, Germany).

## 4. Conclusions

**Figure 9:**
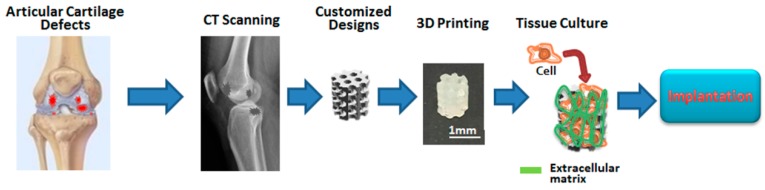
The proposed process of customizing cartilage tissue for cartilage repair in the future. The computerized tomographic (CT) images of the articular cartilage defects are applied to enable the design of the printed scaffolds and three-dimensional reconstruction. The customized porous scaffolds which are similar to the shape of the cartilage defect of the recipient site are designed and printed with water-based light-cured PU/HA hybrid materials by DLP technology. Furthermore, the targeted cells are cultured on the printed PU/HA hybrid scaffolds. After tissue maturation, the cartilage tissues can be used in implantation for cartilage repair.

We report new water-based 3D printing photosensitive materials with HA, applied for the development of 3D printed scaffolds for cartilage tissue engineering with DLP technology. The materials bring out important advantages such as non-toxic, high printing resolution, good cytocompatibility, and environmentally friendly. Besides, the 3D printed scaffolds can facilitate cell adhesion, proliferation, and chondrogenic differentiation. Furthermore, the development may be applied for customized cartilage tissue reconstruction in the future (Figure 9).
